# Characteristics and Outcome of Patients With Dual Pulmonary Tuberculosis and Non-mycobacterial Respiratory Infections

**DOI:** 10.4021/jocmr732w

**Published:** 2011-11-10

**Authors:** Gen-Min Lin, Feng-Yee Chang, Chung-Hsing Chou, Yen-Po Lin, Chih-Hung Ku

**Affiliations:** a Department of Internal Medicine, Hualien Armed Forces General Hospital, Hualien, Taiwan; b Department of Internal Medicine, Tri-Service General Hospital, Taipei, Taiwan; c Department of Medicine, Tzu-Chi General Hospital, Taipei branch, Taiwan; d Department of Public Health National Defense Medical Center, Taipei, Taiwan

## Abstract

**Background:**

Mixed tuberculosis (TB) and bacterial respiratory infections are usually seen in areas where there is an HIV epidemic. However, there have been no previous reports regarding TB patients with concomitant bacterial respiratory infections in a non-HIV prevalent region. This study was to evaluate the clinical characteristics and outcomes of patients with dual TB and bacterial respiratory infections in Hualien, Taiwan.

**Methods:**

A retrospective cohort study was conducted in a community teaching hospital in Hualien from 2000 to 2007. Those who fulfilled the criteria for active pulmonary tuberculosis (PTB) were included and divided into subjects with concomitant bacterial infections and controls. Their basic data, clinical presentations and in-hospital outcomes were reviewed and analyzed.

**Results:**

During 2000 - 2007, a total of 182 patients were diagnosed as having PTB. Of them, 54 (29.7%) had dual infections. Comorbidities were common in these patients. Older age and lower socioeconomic status were present in subjects than in controls. In terms of disease characteristics, symptoms of cough and sputum production, laboratory findings of leukocytosis with left shift, thrombocytopenia, renal insufficiency and lower serum albumin level, as well as radiographic patterns of multi-lobar infiltrates and alveolar consolidations prevailed amongst subjects (P < 0.05). Delayed diagnosis in PTB and increased rates of in-hospital morbidity and mortality associated with polymicrobial infections were noted in subjects with dual infections.

**Conclusions:**

In a non-HIV prevalent area, patients of older age, lacking access to good health care, and suffering from malnutrition were predisposed to dual infections and had poor prognosis and outcomes.

**Keywords:**

Pulmonary tuberculosis; Dual nontuberculous bacterial respiratory infections

## Introduction

Tuberculosis (TB) has been emphasized as a worldwide contagious infectious disease since the emergence of multidrug-resistant strains, an increasing population of human immunodeficiency virus (HIV) co-infection, and most importantly, a lack of public health surveillance system for TB control in developing countries. According to the World Health Organization’s (WHO) annual estimations, the majority of new TB cases are concentrated in South-East Asia, Africa and Western Pacific regions. In 2005, 1.6 million people died of TB, and an additional 200,000 deaths resulted from HIV-associated TB [1].

Taiwan, located in the Western Pacific region, is one of the TB epidemic areas in the world. Among Taiwan’s twenty-three counties and municipalities, Hualien was the leading TB prevalent community, where the rate of incidence averaged 120.0 per 100 000 populations over the past decade [2]. In this community, patients with poor medical compliance, low socioeconomic conditions, and alcoholism were the main contributors to new cases of this disease, rather than those with HIV. As a member of the global village, Taiwan’s government has advanced a policy intending to halve the prevalence and mortality rates of TB within ten years, which corresponds to the targets of the ”Global Plan to Stop TB 2006 - 2015“ launched by WHO [3].

To date, there has been a number of research projects conducted in HIV-dense region to discover the poor prognosis of tuberculous patients coexisting with HIV [4,5], especially those with other microbial infections [6]. However, the clinical outcome of pulmonary TB (PTB) and concurrent bacterial infections has rarely been demonstrated in a non-HIV associated TB-prevalent area. To our knowledge, mycobacterial antigens promote expression of inhibitory cytokines and further depress response of T-helper cells, which results in immunosuppresion, deactivation of macrophage, and disease progression [7,8]. Not only does reduced host immunity expose the tuberculous patient to opportunistic microbial infections, but poor living conditions does so as well. We hypothesize that both the functions of immunity and environmental factors, other than HIV, will determine the fate of patients with concomitant PTB and bacterial infections. The aim of our study is to investigate the clinical characteristics and in-hospital outcomes of patients with dual active PTB and community-acquired non-mycobacterial respiratory infections in Hualien, Taiwan.

## Materials and Methods

### Study subjects

The retrospective cohort study was conducted in Hualien Armed Forces General Hospital, a 400-bed community teaching hospital in eastern Taiwan. The hospitalized patients are mainly composed of military staff, conscripts and their families, veterans, inhabitants of the geriatric facility as well as civilians. Every patient admitted to our pulmonary section on the impression of respiratory tract infections will receive a thorough history taking and physical examinations. Chest roentgenogram, laboratory tests for whole blood cells, and biochemistries were obtained on admission. Routine screen of sputum or pleural fluid cultures for bacteria and *Mycobacterium tuberculosis* and serologic tests for atypical pathogens were carried out after admission. Pleural biopsy and PTB-polymerase chain reaction would be done simultaneously with cultures if PTB was highly suspected. New cases of active PTB could be diagnosed about 30 - 40 persons per year in this hospital. We searched the mycobacterial reporting databases from January 2000 to December 2007. Those who fulfilled the criteria for active PTB were included and divided into subjects with dual bacterial infections and controls with isolated TB infection. The following data was reviewed and analyzed from the medical records: demographic characteristics (age, sex, race, origin), socioeconomic status, comorbidities, symptoms, radiological appearance, bacteriological investigation, laboratory investigations (total blood cell count, liver and renal function parameters, and fasting lipid profiles), HIV status, time lag for diagnosis of PTB and in-hospital outcomes (nosocomial pneumonia, respiratory failure, septic shock and mortality). The Tri-Service General Hospital Institutional Review Board approved the study and waived the requirement for informed consent for this retrospective review of medical records (TSGHIRB09705060).

### Definitions

Active PTB was defined, according to the American Thoracic Society classification, that individuals testing positive for the Tuberculin Skin Test displayed clinical as well as radiographic and/or microbiological evidence of *Mycobacterium tuberculosis* and suffered from PTB at the time of sampling [9]. Community-acquired non-mycobacterial respiratory infections present features of respiratory tract infection or appearance of a consolidation or infiltrations in the chest radiograph that was consistent with acute infection. Microbiological studies uncovered the non-mycobacterial species from cultures of sputum/pleural fluid or positive serologic antibody and urinary antigen tests on admission [10]. Criteria for definite diagnosis of bacterial respiratory infection was made as followed: 1. The culture-confirmed bacteria came from both sputum and the sterile sites including blood, pleural fluid and bronchial tract secretions via bronchoalveolar lavage; 2. Patients who had negative acid-fast stains (AFB) without anti-tuberculous treatment responded well to the prescribed antibiotics within one week; 3. Patients with positive acid-fast stain or mixed bacterial infections were excluded. Low socioeconomic status was judged by the patient’s educational attainment (less than a high school degree), poverty (definition by a family’s total income was less than the family’s threshold), or unemployment [11]. Renal insufficiency involved a serum blood urea nitrogen (BUN)/creatinine ratio > 20, a creatinine level > 2.0 mg/dL or a 24-hour urinary creatinine clearance rate < 60 mL/minute. Delayed diagnosis of PTB referred to either initially missed diagnosis of PTB within the first 24 hour after admission mostly owing to absence of associated symptoms, atypical radiographic presentations and unavailable laboratory evidence for TB or delayed antituberculous treatment until 7 days or more if all AFB were negative [12].

### Microbiological studies

Routine microbiological evaluations contained the following tests: two samples for blood aerobic and anaerobic conventional cultures if the patient was febrile on admission; respiratory tract secretions or pleural fluid were utilized for Gram stain/bacterial cultures at once and AFB/mycobacterial cultures for three consecutive days, when specimens were available. Serum was collected for atypical pathogens survey including *Mycoplasma pneumoniae*, *Chlamydophila pneumoniae* and *Legionella pneumophila* antibody measurements: Acute *M. pneumoniae, C. pneumoniae* and/or L. pneumophila infection was diagnosed if the patient had a significant antibody response to one of the pathogens (IgM antibody, a 4-fold increase in IgG antibody titre, a static IgG antibody titre four times or more than the cut-off of the assay). Besides, *Legionella* urine antigen was also used for detecting *L. pneumophila* infection.

### Radiological studies

Serial chest X-rays (CXRs) obtained on admission and during hospitalization were reviewed by a pulmonologist and a radiologist. If a discrepancy existed in the interpretations, the CXR was further reviewed by another chest or radiological specialist blinded to the results. The predominant radiographic pattern (upper lobe infiltrate, multi-lobar infiltrates, alveolar consolidation, pleural effusion, bronchiectasis, and emphysematous change), and the presence of cavities were recorded.

### Statistical analysis

Data was expressed either as percentage for the group (categorical variables) or as mean ± SD (continuous variables). Continuous variables were compared with use of Student’s t-test for normally distributed data. Categorical variables were compared with use of the χ^2^ and Fisher’s exact tests. The statistical package (SAS version 9.1.3, SAS Institute; Cary, NC) was used for all analyses. All P values were 2 sided; P < 0.05 was considered significant. Survival curves were obtained using Kaplan-Meier method and compared with use of log rank tests.

## Result

### Patient characteristics

A total of 182 patients with confirmed PTB accounted for 4.6% of all patients with community-acquired respiratory infections admitted to the hospital within the study period. The baseline characteristics of patients with isolated PTB (controls) and dual infections (subjects) were shown in Table 1. Of them, 54 (29.7%) had concurrent non-mycobacterial respiratory infections at admission. The median age of the 182 patients who enrolled in the study was 53 years (range 16 - 93) and 78.7% (150/182) of the patients were male. The subjects exhibited an older median age than controls (64.0 years versus 48.4 years, P < 0.0001). Indigenes accounted for more than half of hospital admissions (58.8%). No statistically significant differences were observed between the two groups with respect to their origins: geriatric members (31.3% versus 46.3%), military staff and conscripts (20.3% versus 11.1%) and civilian (48.4% versus 42.6%). Over sixty percent of active PTB patients were classified in a low socioeconomic status, particularly in subjects (55.5% versus 74.1%, P = 0.0187). Comorbid illness was common in patients with active PTB, with 51.6% being ever-smokers, 22.5% having COPD, 19.2% having recurrent PTB, 33.5% being alcoholism, 5.5% having hepatitis C virus (HCV) infection, 13.2% having liver cirrhosis, 11.0% having type 2 diabetes and 6% having malignancy. Only one patient (0.5%) was identified as having HIV.

**Table 1:** Patient Characteristics Comparing Groups of Control and Subject

	**Control**		**Subject**		**Total**		**P-value**
		
	**N**	**%**	**N**	**%**	**N**	**%**	
Subjects	128	70.3	54	29.7	182	100	
Age years	48.4 ± 23.0 (20 - 93)		64.0 ± 23.0 (16 - 89)		53.0 ± 24.0 (16 - 93)		< 0.0001
< 30 years	44	34.4	10	18.5	54	29.7	
30-60 years	43	33.6	5	9.3	48	26.4	
> 60 years	41	32.0	39	72.2	80	43.9	
Male sex	106	79.2	44	77.3	150	78.7	NS
Indigenes	80	62.5	27	50.0	107	58.8	NS
Origin							
Geriatric members	40	31.3	25	46.3	65	35.7	NS
Military conscripts	26	20.3	6	11.1	32	17.6	NS
Civilian	62	48.4	23	42.6	85	46.7	NS
Low socioeconomic	71	55.5	40	74.1	111	61.0	0.0187
Comorbidities							
Ever-smoker	64	50.0	30	55.6	94	51.6	NS
COPD	28	21.9	13	24.1	41	22.5	NS
Recurrent PTB	27	21.1	8	14.8	35	19.2	NS
Alcoholism	44	34.4	17	31.5	61	33.5	NS
HCV	5	3.9	5	9.3	10	5.5	NS
Cirrhosis	15	11.7	9	16.7	24	13.2	NS
Renal insufficiency	13	10.2	15	27.8	28	15.4	0.0041
Diabetes	13	10.2	7	13.0	20	11.0	NS
Malignancy	5	3.9	6	11.1	11	6.0	NS
HIV	0	0	1	1.9	1	0.5	NS

Data are presented as mean ± SD (range) unless otherwise indicated. NS: non-significant; PTB: pulmonary tuberculosis; COPD: chronic obstructive pulmonary disease; HCV: hepatitis C virus; HIV: human immunodeficiency virus. P-value for comparisons between PTB and dual infections.

### Disease presentations

Table 2 illustrates clinical symptoms, laboratory, and radiographic characteristics of patients with active PTB on admission. Cough and sputum production were the most common symptoms (81.9%) and there was a statistically significant difference between these two groups (78.1% versus 90.7%, P = 0.0436). In laboratory investigations, significantly higher white blood cell counts (WBC) (11.7 ± 5.2 x 10^3^/μL versus 9.0 ± 4.3 x 10^3^/μL, P = 0.0005) with left shift and lower platelet counts (259.0 ± 108.5 x 10^3^/μL versus 302.1 ± 134.5 x 10^3^/μL, P = 0.0400) were seen in subjects on admission compared with controls. A number of biochemical parameters of serum showed no statistically significant differences between the two groups including electrolytes: sodium (Na) and potassium (K), creatinine, hepatobiliary profiles: aspartate aminotransferase (AST), alanine aminotransaminase (ALT), total bilirubin, gamma glutamyl transpeptidase (γ-GT), and alkaline phosphatase, as well as lipid profiles: total cholesterol and triglyceride. There were statistically significant differences between the two groups with respect to the serum BUN (15.0 ± 8.8 mg/dL versus 25.3 ± 21.4 mg/dL, P = 0.0014) and albumin (3.1 ± 0.7 mg/dL versus 2.8 ± 0.8 mg/dL, P = 0.0091).

**Table 2:** Symptoms, Laboratory and Radiographic Characteristics of Active PTB Patients

	Control		Subject		Total		P-value
		
	N	%	N	%	N	%	
Clinical symptoms							
Fever	68	53.1	23	42.6	91	50.0	NS
Cough/sputum	100	78.1	49	90.7	149	81.9	0.0436
Hemoptysis	13	10.2	7	13.0	20	11.0	NS
Chest pain	18	14.1	6	11.1	24	13.2	NS
Weight loss	24	18.8	7	13.0	31	17.0	NS
Laboratory characteristics							
WBC (10^3^/L)	9.0 ± 4.3		11.7 ± 5.2		9.8 ± 4.7		0.0005
Hemoglobin (g/dL)	12.3 ± 1.8		11.8 ± 2.1		12.2 ± 1.9		NS
Platelet (10^3^/L)	302.1 ± 134.5		259.0 ± 108.5		288.7 ± 128.2		0.0400
Neutrophil (%)	74.0 ± 11.3		78.4 ± 13.7		75.4 ± 12.2		0.0318
Lymphocyte (%)	16.7 ± 8.8		12.6 ± 8.3		15.4 ± 8.8		0.0059
Na (mmol/L)	134.7 ± 5.2		133.2 ± 7.6		134.3 ± 6.0		NS
K (mmol/L)	4.0 ± 0.6		3.9 ± 0.7		4.0 ± 0.6		NS
BUN (mg/dL)	15.0 ± 8.8		25.3 ± 21.4		18.1 ± 14.6		0.0014
Creatinine (mg/dL)	1.0 ± 0.6		1.2 ± 0.8		1.1 ± 0.6		NS
AST (U/L)	39.0 ± 37.1		52.2 ± 61.5		42.7 ± 45.5		NS
ALT (U/L)	29.1 ± 28.0		50.7 ± 121.0		34.9 ± 67.2		NS
Total bilirubin (mg/dL)	1.1 ± 1.3		1.1 ± 0.9		1.1 ± 1.2		NS
G-GT (U/L)	65.5 ± 103.2		91.2 ± 174.1		72.3 ± 125.7		NS
Alkaline phosphatase (U/L)	93.7 ± 48.3		90.0 ± 47.2		92.3 ± 47.7		NS
Cholesterol (mg/dL)	128.1 ± 39.1		121.1 ± 53.4		126.1 ± 43.5		NS
Triglyceride (mg/dL)	89.6 ± 57.9		85.7 ± 59.6		88.4 ± 58.2		NS
Albumin (mg/dL)	3.1 ± 0.7		2.8 ± 0.8		3.0 ± 0.8		0.0091
Radiographic characteristics							
Upper lobe infiltrate	70	54.7	18	33.3	88	48.4	0.0085
Multi-lobar infiltrates	11	8.6	12	22.2	23	12.6	0.0115
Alveolar consolidation	33	25.8	26	48.2	59	32.4	0.0032
Pleural effusion	24	18.8	8	14.8	32	17.6	NS
Cavitary lesion	13	10.2	3	5.6	16	8.8	NS
Bronchiectasis	8	6.3	3	5.6	11	6.0	NS
Emphysema	20	15.6	4	7.4	24	13.2	NS

Data are presented as mean ± SD and N (%) unless otherwise indicated. NS: non-significant; PTB: pulmonary tuberculosis; WBC: white blood cell counts; Na: sodium; K: potassium; BUN: blood urea nitrogen; AST: aspartate aminotransferase; ALT: alanine aminotransaminase; Gamma-GT: gamma glutamyl transpeptidase.

As to radiological presentations on admission, classical upper lobe infiltrate was more prominent in controls, but alveolar consolidation and multi-lobar infiltrative patterns were more extensive in subjects. Pleural effusion, cavitation, bronchiectasis and emphysematous change were equivalent between these two groups.

### Acid-fast stain, delayed diagnosis of active PTB and in-hospital outcomes

**Figure 1:**
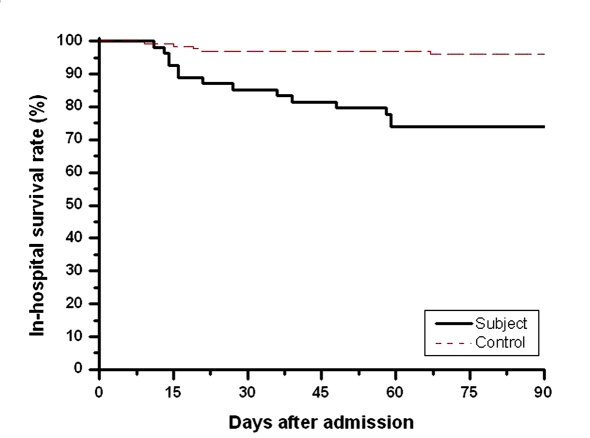
In-hospital survival curves of subjects (black line) and controls (red line) using Kaplan-Meier. P (log rank test) < 0.005.

As shown in Table 3, the subjects had a higher incidence in the delayed diagnosis of active PTB than controls (46.3% versus 28.8%, P = 0.0271) despite they had similar rates of positive AFB. Statistically significant differences existed between the two groups with respect to in-hospital outcomes including nosocomial pneumonia (7.8% versus 22.2%, P = 0.0064), acute respiratory failure (8.6% versus 15.4%, P < 0.0001), septic shock (3.1% versus 14.8%, P = 0.0069), and mortality (3.9% versus 25.9%, P < 0.0001). The mean in-hospital survival of the subjects who died was 31.6 days (range 11 - 59) with 50% of them dying within the first 21 days and 75% of them dying within the first 48 days (Fig. 1).

**Table 3:** Sputum AFB, Delayed Diagnosis of PTB and In-hospital Outcomes of Both Groups

	Control		Subject		Total		P-value
		
	N	%	N	%	N	%	
Positive sputum AFB	61	47.7	28	51.9	92	50.5	NS
Delayed diagnosis of PTB	37	28.8	25	46.3	62	34.1	0.0271
In-hospital outcomes							0.0436
Nosocomial pneumonia	10	7.8	12	22.2	22	12.1	0.0064
Acute respiratory failure	11	8.6	17	31.5	28	15.4	< 0.0001
Septic shock	4	3.1	8	14.8	12	6.6	0.0069
Mortality	5	3.9	14	25.9	19	10.4	< 0.0001

Data are presented as N (%) unless otherwise stated. NS: non-significant; AFB: acid-fast stain; PTB: pulmonary tuberculosis.

### Microbiological characteristics

In mycobacterial investigations, only one subject had miliary TB, which was proved by liver biopsy and another two had multi-drug resistant TB (MDR-TB) during the period of anti-tuberculous treatment in the group of dual infections (data not shown). Table 4 demonstrated the bacterial species and the associated number of the subjects. A total of 20 subjects with atypical bacterial infections: 12 having *M. pneumoniae*, 6 having *L. pneumophila*, and 2 having *C. pneumoniae*. There were 13 subjects with Gram-positive bacterial infections: 6 having *Steptococcus pneumoniae*, 6 having *Staphylococcus aureus*, and 1 having *Enterococcus species*. Additionally, 33 subjects with Gram-negative bacterial infections were reported: 11 having *Klebsiella pneumoniae*, 6 having *Haemophilus influenzae*, 4 having *Pseudomonas aeruginosa*, 4 having *Escherichia coli*, 3 having *Proteus mirabilis*, 3 having *Stenotrophomonas maltophilia* and 2 having *Enterobacter*. Only 20 subjects meet criteria for definite dual respiratory infections. Table 5 displayed an equivalent mortality rate of the subjects with different kinds of bacterial infections (atypical versus Gram-positive versus Gram-negative: 30.0% versus 30.8% versus 30.2%). However, we noticed that the subjects with poly-bacterial infections had a higher mortality rate than those with mono-bacterial infections (43.5% versus 12.9%, P = 0.0147).

**Table 4:** Bacterial Species and the Associated Number of the Subjects

Bacterial species	Number (N)
Atypical pathogens	
* Mycoplasma pneumonia*	12
* Legionella pneumonia*	6
* Chlamydia pneumnia*	2
Gram-positive pathogens	
* Steptococcus pneumonia*	6
* Staphylococcus aureus*	6
* Enterococcus species*	1
Gram-negative pathogens	
* Klebsiella pneumoniae*	11
* Haemophilus influenzae*	6
* Pseudomonas aeruginosa*	4
* Escherichia coli*	4
* Proteus mirabilis*	3
* Stenotrophomonas maltophilia*	3
* Enterobacter*	2

**Table 5:** Microbiological Characteristics and the Prognosis in Patients With Dual Infections

	Survivors		Deceased		Total		P-value
		
	N	%	N	%	N	%	
Bacteriological classifications							NS
Atypical bacteria	14	70.0	6	30.0	20	30.3	
Gram-positive bacteria	9	69.2	4	30.8	13	19.7	
Gram-negative bacteria	23	69.7	10	30.2	33	50.0	
Number of non-TB bacterial infections							0.0147
Poly-bacterial infection	13	56.5	10	43.5	23	42.6	
Mono-bacterial infection	27	87.1	4	12.9	31	57.4	

Data are presented as N (%) unless otherwise stated. NS: non-significant; TB: tuberculosis.

## Discussion

Based on the investigations in Hualien by Taiwan’s Centers for Disease Control (CDC) over the past 8 years, 80% of all tuberculous patients were PTB and 70% of the new cases were male. At the time of diagnosis, the mean age for patients ranged from 52 to 55 year-old and almost 50% were elderly, or those who were more than 65 years in age. Ethnic minorities (indigenes) had higher incidence than the ethnic majorities [2,13]. Military conscripts have also shown high prevalence of TB on previous reports [14,15]. In our study, recognized PTB patients of the hospital shared similar demographic characteristics with those in the general survey of Hualien. Smoking, alcoholism, and chronic organic diseases were most common among these people. On the contrary, HIV infections were encountered in only 0.5% of patients with TB and the prevalence rate was close to the nationwide statistic [13]. The main findings were that people with older age and low socioeconomic status were predisposed to concomitant active PTB and non-myobacterial respiratory infections. As we know, aging itself had the natural history of progressive immune dysregulation such as a reduction in CD8+ T cells and a decline in T cell proliferation [16,17]. These factors accounted for not only the increased rates of TB, but also non-mycobacterial infections in the elderly. Second, low socioeconomic status has been a well-known risk of *M. tuberculosis* infection, but such a connection was absent for other non-myobacterial pathogens [18,19]. The reason for that was probably because aged persons represented were of low socioeconomic status in Hualien; with the elderly proportionally increasing in number, low socioeconomic status reflected a higher rate in the group of dual PTB and bacterial infections than in control.

It was not surprising that patients with dual infections presented more frequent symptoms of cough and sputum production, leukocytosis with left shift, and less platelet counts that were caused by the host immune response to the external stimuli of pathogenic bacteria. Regarding to the laboratory finings of renal insufficiency with elevating BUN level and lower serum albumin in subjects, these implied the underlying malnutrition, status of volume depletion, and advanced illness [20-21]. With the accumulated evidence, malnourished people with impaired immunity were vulnerable to *M. tuberculosis* and other microbial infections [22,23]. Recently, numerous studies showed that a lower serum albumin level obtained on admission could be used as an independent risk of in-hospital death among patients with active TB [24,25]. With respect to the radiological appearances at admission, patterns of multi-lobar infiltrates and alveolar consolidation rather than classical upper lobe lesions of tuberculosis were mostly seen in subjects. It correlated well with the radiological presentations of PTB in elderly and superimposed inflammatory process conferred by non-mycobacterial infections [26,27]. These unexpected expressions of double pulmonary infections clinically masked active PTB and further delayed the diagnosis of tuberculosis until the cultures were available. As compared to the two groups with active PTB, more in-hospital morbidity and mortality rates were observed in the group of dual infections. A number of variables other than lower serum albumin level mentioned above have been acknowledged as risks of adverse outcomes in PTB patients, including older age, more extensive infiltrative and consolidation patterns on chest radiographs, and delayed anti-tuberculous treatment [28-29]. Obviously, more risks imposed on patients with dual infections could lead to poor clinical outcomes.

In analyzing the bacteriologic characteristics, dual PTB and bacterial respiratory infections were remarkable (54/182, 29.7%) in this non-HIV epidemic area. This may reflect the age, general health and socioeconomic status of the patients as well as the criterion we used for non-mycobacterial respiratory tract disease (20/182, 11.0% for definite diagnosis). Whether a strict selection for subjects was taken or not, an association between atypical pathogens and environment was present. We found the relatively young age of military conscripts and students were chief sufferers of *M. pneumoniae* and elderly geriatrics accounted for *C. pneumoniae* and *L. pneumophila*. The other kinds of bacteria species were mainly dependent on the age and the related comorbidities of patients regardless of the place. The elderly with limited activity were prone to have Gram-positive and Gram-negative bacterial infections. The retrospective analysis of these 54 case patients found an in-hospital mortality rate of 25.9%. There had some reports describing such a high mortality rate among HIV patients with active TB and specific bacteria like *L. pneumophila* and *S. pneumoniae* infections [30,31]. In addition, hospital-acquired gram-negative bacterial pneumonia has also been regarded as a poor prognostic factor for patients with TB [32]. However, this correlation was not appropriate for our PTB patients with community-acquired bacterial respiratory infections due to the limited case numbers, less drug-resistant strains, and no difference in mortality rate regarding to bacteriological classifications. In our observations, poly-bacterial infections occurred in 42.6% of subjects and had a significantly higher mortality rate than that of mono-bacterial infection. Although poly-bacterial respiratory infections could result from benign microbial colonization leading to the incidence rate overestimated, they were pathogenetically associated with poor oral hygiene, aspirations, diabetes mellitus, specific viral co-infection and extensive immunosuppression [33-35]. Under these circumstances, patients presented more severe illness on admission, which may cause the following high morbidities and mortalities in hospital.

In conclusion, concomitant TB and bacterial respiratory infections were prevalent in Hualien, Taiwan. The majority of patients were elderly with lower socioeconomic status. Comobidities were common in this population. Patients presented more symptoms of cough with sputum production, and laboratory findings of leukocytosis with left shift, thrombocytopenia, renal insufficiency and lower serum albumin level. Besides, multi-lobar infiltrates and alveolar consolidations patterns were predominant in chest radiograph at admission. Delayed diagnosis in active PTB as well as increased rates of in-hospital morbidity and mortality related to poly-bacterial infections were observed.

The main limitation was that dual infections could not be convinced depending on either presumptive definition or definite criteria. We may overestimate the “false subjects” with benign bacterial colonization by loosely presumptive definition and underestimate the “true subjects” with positive AFB receiving successful combined antibiotics and anti-tuberculous treatment by definite criteria. Nevertheless, the study clarified the factors contributing to dual TB and bacterial respiratory infections and the associated outcomes in Hualien, Taiwan. A better geriatric health care policy to reduce the overall mortality of PTB in a non-HIV associated TB prevalent area was needed and should be worked out immediately.
